# The identification of the TRPM8 channel on primary culture of human nasal epithelial cells and its response to cooling

**DOI:** 10.1097/MD.0000000000007640

**Published:** 2017-08-04

**Authors:** Shao-Cheng Liu, Hsuan-Hsuan Lu, Hueng-Chuen Fan, Hsing-Won Wang, Hang-Kang Chen, Fei-Peng Lee, Chong-Jen Yu, Yueng-Hsiang Chu

**Affiliations:** a Department of Otolaryngology-Head and Neck Surgery, Tri-Service General Hospital, National Defense Medical Center; b Graduate Institute of Clinical Medicine, College of Medicine, Taipei Medical University; c Department of Internal Medicine, National Taiwan University Hospital, College of Medicine, National Taiwan University; d Department of Pediatrics, Tungs’ Taichung Metro Harbor Hospital; e Department of Otolaryngology-Head and Neck Surgery, Shuang Ho Hospital, Taipei, Taiwan, Republic of China.

**Keywords:** cold receptor, immunohistochemistry, menthol, mucin, TRPM8

## Abstract

**Background:**

It has been proposed that the transient receptor potential (TRP) channel Melastatin 8 (TRPM8) is a cold-sensing TRP channel. However, its presence and its role in the nasal cavity have not yet been fully studied.

**Methods:**

Immunohistology was used to study TRPM8 receptors in both the nasal mucosa tissue and the primary cultures of human nasal cells. Cells from primary cultures were immunostained with antibodies to TRPM8, mucin, cytokeratin (CK)-14, CK-18, and vimentin. Western blotting and real-time polymerase chain reaction (PCR) were used to determine the physiological role of TRPM8 in mucus production in the nasal cavity, with and without its agonist and antagonist.

**Results:**

The TRPM8 is clearly present in the epithelium, mucous glands, and vessels. No obvious TRPM8-immunoreactive cells were detected in the connective tissue. Immunostaining of cytospin preparations showed that epithelial cells test positive for CK-14, CK-18, TRPM8, and mucin 5AC (MUC5AC). Fibroblastic cells are stained negative for TRPM8. Secreted mucins in the cultured supernatant are detected after exposure to menthol and moderate cooling to 24°C. Both induce a statistically significant increase in the level of MUC5AC mRNA and mucin production. BCTC, a TRPM8 antagonist, has a statistically significant inhibitory effect on MUC5AC mRNA expression and MUC5AC protein production that is induced by menthol and moderate cooling to 24°C.

**Conclusions:**

The study demonstrates that TRPM8 is present in the nasal epithelium. When it is activated by moderate cooling to 24°C or menthol, TRPM8 induces the secretion of mucin. This study shows that TRPM8 channels are important regulators of mucin production. Therefore, TRPM8 antagonists could be used to treat refractory rhinitis.

## 1 Introduction

The transient receptor potential (TRP) channels superfamily are nonselective cation channels and can be classified into 6 related subfamilies: M (melastatin, TRPM), C (canonical, TRPC), V (vanilloid, TRPV), A (ankyrin, TRPA), P (polycystin TRPP), and mucolipin.^[1]^ Of these TRP subfamilies, melastatin 8 (TRPM8) is activated by cold temperatures and menthol^[2]^ and is responsible for the detection of a cold environment.^[3,4]^ Breathing cold air can cause coughing, bronchial constriction, and mucosal secretion; and these responses can develop into an asthma attack or may exacerbate preexisting chronic obstructive pulmonary diseases (COPD). In the literature, the TRPM8 channel is expressed in human bronchial epithelial cells.^[5–7]^ Cold temperatures also induce the mucus hypersecretion from normal human bronchial epithelial cells in vitro, through TRPM8 activation.^[8]^ The physiological function of the nasal cavity is to condition the air that enters by warming and humidifying the inhaled cold air. In healthy subjects, cold air induces significant rhinorrhea.^[9]^ Menthol, a TRPM8 agonist, induces calcium influx and the release of histamine in cell line preparation, which suggests that TRPM8 has a role in histamine-driven allergic rhinitis.^[10]^ However, its presence and the role of this TRPM8 “cold receptor” in nasal mucosa is not fully understood.

Previously, researchers in the field of respiration have been hindered by lack of a reliable experimental model. Most studies of respiration use primary cultures of the lower airway^[8]^ to study biological mechanisms. The upper airway, especially in primary culture study, has not been fully studied. To determine the pathophysiological role of cold in human nasal mucin production, primary cultures of human nasal epithelial (HNE) cells were established in this study, to determine the level of TRPM8 expression in different components of human nasal mucosa tissue, to study TRPM8 expression in a primary culture of HNE cells from patients with allergic rhinitis, and to determine the validity of the hypothesis that cold-mediated activation of the TRPM8 channel is responsible for the enhanced expression of mucin 5AC (MUC5AC), which is one of the major airway mucins that is implicated in chronic airway diseases with mucus hypersecretion.

## 2 Materials and methods

### 2.1 Human nasal tissue preparation and primary culture

All cell culture reagents were purchased from Sigma-Aldrich (St. Louis, MO), unless otherwise stated. Nasal mucosa specimens were prepared from inferior turbinate samples after a partial turbinectomy on 8 patients (6 men and 2 women) who suffered from chronic nasal congestion due to turbinate hypertrophy or allergic rhinitis. The specimens were rinsed with Dulbecco phosphate-buffered saline (PBS). A section of each specimen was preserved for immunohistochemical staining. The remainder of the tissues were dissociated with dispase II solution at 37°C for 1 hour and then immersed in PBS, before dissection. To obtain the epithelium cells, toothed forceps were used to scratch the surface of a nasal mucosa specimen. The dissociated cells were collected and the supernatant PBS solution was removed by centrifugation. An adequate amount of trypsin/EDTA (0.125%) was added and the tube was incubated in water at 37°C for 15 min and vortexed every 5 min. Dulbecco modified eagle medium (DMEM) with 10% fetal bovine serum (FBS) was then used to neutralize the activity of trypsin and was removed after centrifugation. The cells were then suspended in keratinocytes serum-free medium (KSFM) and cultured in a 25 mL flask. The remainder of the specimens without epithelium cells were cut into small pieces and dissociated with collagenase I (3 mg/mL) at 37°C for 1 hour. DMEM was then used to neutralize it and to remove the medium after centrifugation. Finally, dissociated fibroblastic cells were suspended in DMEM and cultured in a 25 mL flask. Both types of cells were cultured in a humidified chamber containing 95% air and 5% CO_2_ at 37°C. The morphological features of the cells were determined and recorded every day using an inverted light microscope (Olympus, Shinjuku-ku, Tokyo). Growth rates (cells/d/cm^2^) for the cultured epithelial and fibroblast cells were calculated and recorded at every passage.

### 2.2 Immunohistochemistry

Nasal turbinate tissue from the 8 patients were fixed in 4% buffered formalin for at least 24 hours and then processed in paraffin. Serial 4 μm paraffin sections were prepared and the paraffin removed. Endogenous peroxidase was blocked with 3% H_2_O_2_ in methanol/PBS (1:1 ratio) for 10 min, followed by 30 min incubation with 2% horse serum. After a brief rinse, the sections were subjected to antigen retrieval by heating the slides to 56°C in a sodium citrate buffer for 30 min. The slides were then rinsed in PBS, followed by a 90 min incubation with TRPM8 monoclonal antibody (Abcam, Cambridge, UK). The remainder of the steps and the antigen staining was performed according to the manufacturer's instructions (Thermo Scientific, Waltham, MA). For the negative control, the slide was incubated without primary antibody. The slides were then examined under the microscope and the images, each of which contained the morphological structures with recognizable positive staining, were subjected to semiquantitative scoring. The values used were 0 points for negative, 1 point (+) for slightly positive, 2 points (++) for clearly positive, and 3 points (+++) for extremely positive staining. All data were averaged and rounded.

Cells from primary cultures were trypsinized, the cells suspension was prepared in PBS, and cytocentrifugation was performed at 1500 rpm for 5 min, to allow the cells to bind to the glass slide. The cytospin slides were immunostained (ABC immunoperoxidase) with antibodies to TRPM8, mucin, cytokeratin (CK)-14, CK-18, and vimentin, as described previously.^[11,12]^ The specificity of the antibody was ensured by omitting the primary antibody in control sections.

### 2.3 Determination of MUC5AC mRNA expression and protein level

Menthol, a TRPM8 agonist, and N-(4-tert-butylphenyl)-4-(3-chloropyridin-2-yl) piperazine-1-carboxamide (BCTC), the antagonists of TRPM8, were used as the testing agents. There were 5 groups: the control, menthol at 10^−4^ M, BCTC (10^−4^ M), menthol and BCTC, and cold condition at 24°C. These agents were tested with a 4-hour exposure. All cells, except the cold condition group, were cultured at 37°C.

Exon 18-specific TRPM8 small hairpin RNA (shRNA), and scramble shRNA were purchased from Santa Cruz Biotechnology (#sc-95009) and were transfected into the HNE cells according to the manufacturer's instruction. The experiments were repeated according to the aforementioned protocol, using HNE cells with/without TRPM8 or scramble shRNA transfections. The MUC5AC mRNA transcripts were measured using real-time quantitative polymerase chain reaction (PCR). The PCR primers for human MUC5AC were designed according to the published cDNA sequences: forward 5′ GCTCAGCTGTTCTCTGGATGAG 3′; reverse 5′ TTACTGGAAAGGCCCAAGCA 3′.

The production of MUC5AC protein in culture supernatants was measured by western blot analysis. Cell culture supernatants were collected after elimination, filtration, and concentration, using Amicon Ultra 100K centrifugal filter devices (Millipore). After adjustment according to the HNE cell counts for each group, proper protein samples were loaded onto a 6% SDS-PAGE gel and transferred to polyvinylidene difluoride (PVDF) membranes. After blocking with 5% nonfat milk in Tris-buffered saline with Tween, the membrane was incubated overnight with primary antibodies that are specific to the MUC5AC monoclonal antibody (mouse anti-MUC5AC, Thermo Scientific No. MA5-12178, 1:250). Subsequently, the membrane was incubated with the appropriate secondary antibody for 1 hour and immunoreactive bands were visualized using a chemiluminescence Pierce ECL kit (Thermo Scientific, Waltham, MA). The relative band intensity was quantified using TotalLab Quant software (TotalLab Ltd, Newcastle, UK).

*Statistical analysis:* The data is presented as mean values and SD. The differences between mean values were compared using a Student *t* test, and were assumed to be significant at *P* < .05.

*Ethical considerations:* The research protocol was reviewed and approved by the Institutional Review Board of the Tri-Service General Hospital.

## 3 Results

### 3.1 Cells in primary culture

**Figure 1:**
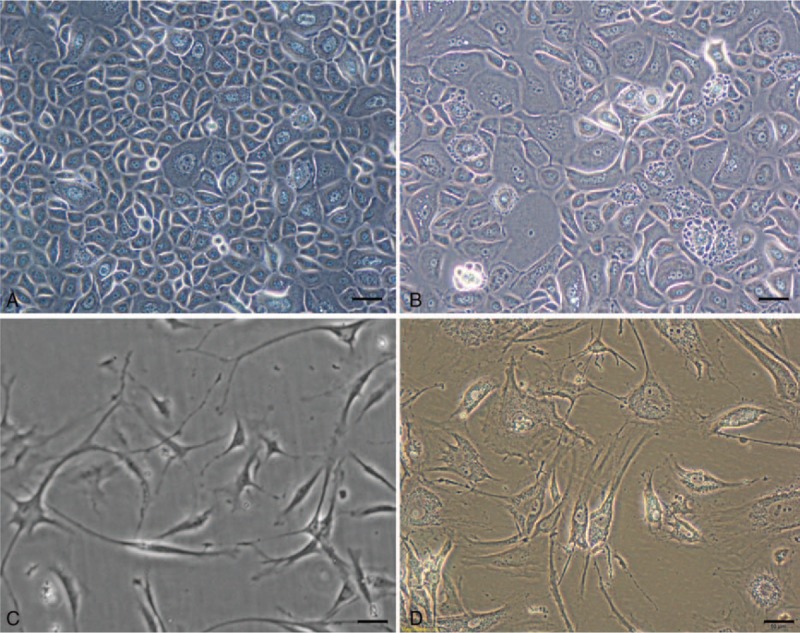
A micrograph of cultured cells at a magnification of 200X: (A) at passage 1, showing the typical cobblestone morphology of epithelial cells, (B) epithelial cells begin to swell at passage 6, (C) at passage 1, fibroblastic cells are elongated (spindle-shaped) cells with a branched cytoplasm surrounding an elliptical nucleus, and (D) cells are swollen at passage 7.

A primary culture of HNE cells was successfully established using the inferior turbinate samples from the 8 patients. Cultured HNE cells are morphologically distinguishable from the fibroblastic cells. Epithelium cells have a polygonal cobblestone appearance with clearly identified nuclei and granules within the cytoplasm (Fig. 1A). Fibroblastic cells are large, flat, and elongated (spindle-shaped) cells with processes extending from the cell body, with a branched cytoplasm surrounding an elliptical, speckled nucleus that has 2 or more nucleoli (Fig. 1C). Both epithelium and fibroblastic cells that were cultured retain the polygonal morphology from the primary culture up to passage 5. After that, the cells begin to swell (Fig. 1B and D).

**Figure 2:**
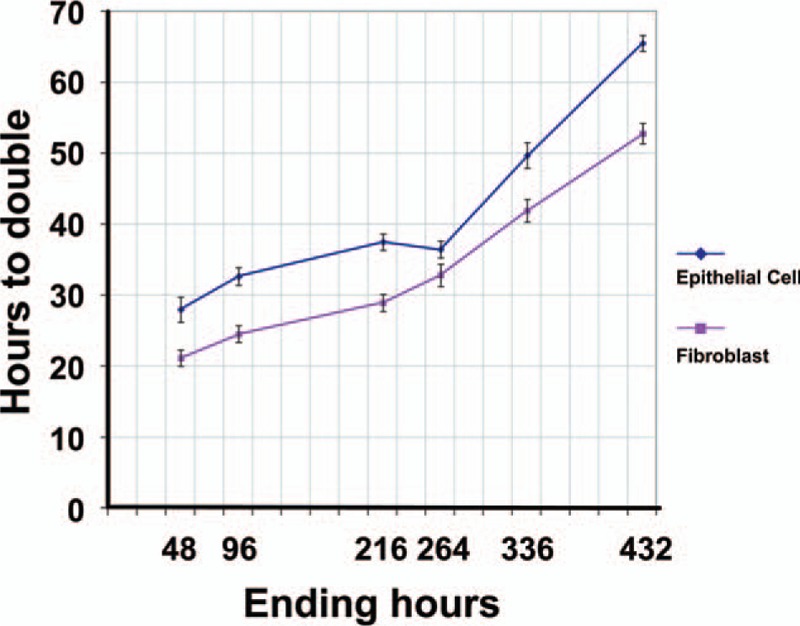
PDT of epithelium and fibroblastic cells. Fibroblastic cells show a higher growth profile than epithelial cells. PDT = population double time.

For epithelium cells, the growth rate (population double time [PDT]) is 27.93 ± 1.77 hours at passage 1 (P1). At P2, the PDT is extended to 32.59 ± 1.26 hours. The PDT is extended at each passage. At P3, P4, P5, and P6, the PDT is 37.45 ± 1.15, 36.42 ± 1.19, 49.68 ± 1.81, and 65.44 ± 1.11 hours, respectively. Fibroblastic cells show a higher growth profile than epithelial cells. The PDT is 21.11 ± 1.13 hours at P1 and 24.49 ± 1.16 hours at P2. Similarly, the growth rate slows daily and the PDT is 28.88 ± 1.21, 32.77 ± 1.55, 41.86 ± 1.63, and 52.73 ± 1.46 at P3, P4, P5, and P6, respectively (Fig. 2).

### 3.2 Immunohistochemistry

**Figure 3:**
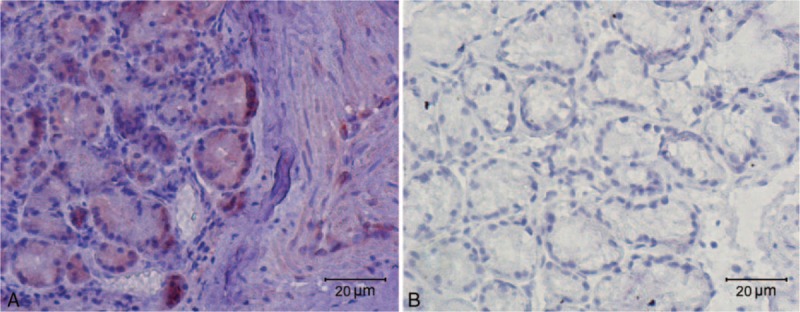
A micrograph of human nasal tissue at 400X magnification: (A) TRPM8 immunostaining is present principally in the epithelium, secretory gland, and vessels, with no obvious TRPM8-immunoreactive cells detected in the connective tissue and (B) the negative control.

TRPM8 is present in epithelium, mucous glands, and vessels (Fig. 3). The average values for the semiquantitative scoring for TRPM8 staining are 2.13, 1.75, and 1.88 points in epithelium, vessels, and glands, respectively (Table 1). No obvious TRPM8-immunoreactive cells are detected in the connective tissue.

**Table 1:**
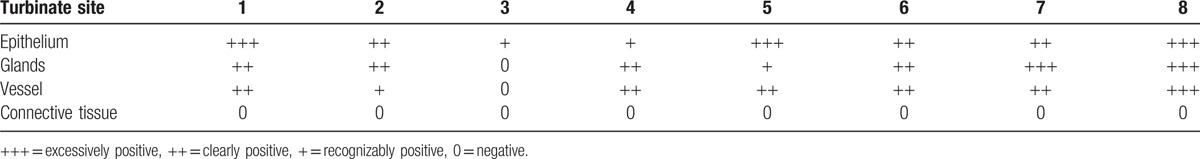
Semiquantitative scores for the staining of TRPM8 in human turbinate mucosa.

**Figure 4:**
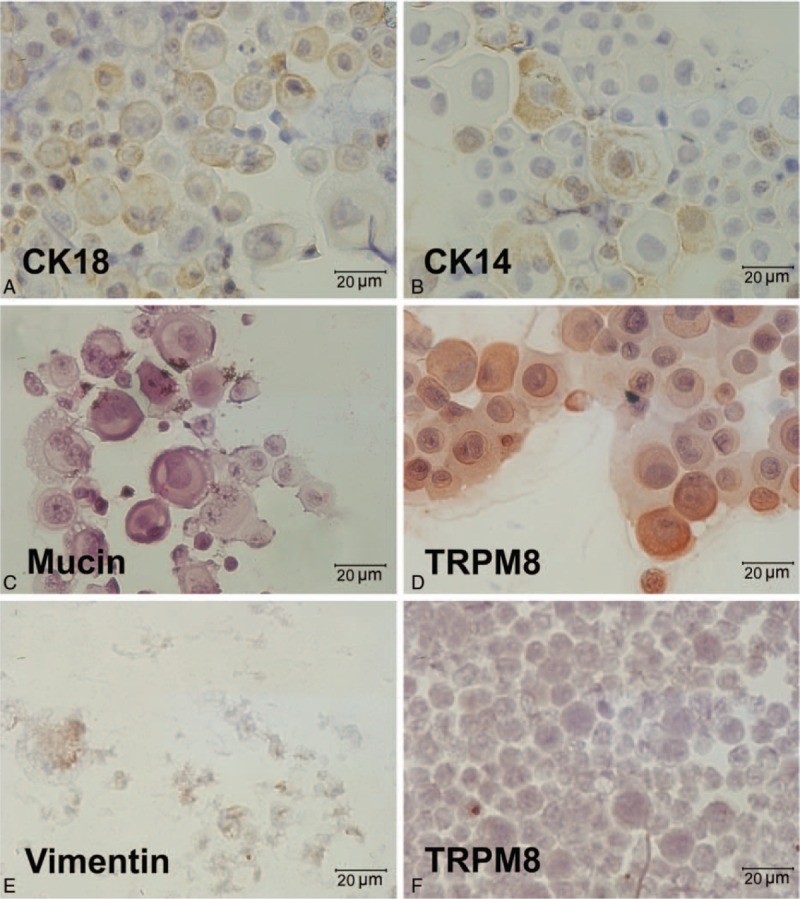
Immunocytochemistry for primary culture cells. Epithelial cells (A–D) are positive for CK18, CK14, mucin, and TRPM8. Fibroblastic cells are positive for vimentin (E) but negative for TRPM8 (F). (Original magnification: ×400).

Immunostaining of epithelial cells is positive for CK-14 (cytoskeleton of epithelial cells), CK-18 (secretory epithelial cells), TRPM8 (cold receptor), and MUC5AC (mucins in the airway) (Fig. 4 A–D). Fibroblastic cells are stained positive for vimentin (mesenchymal marker) but negative for keratin and TRPM8 (Fig. 4 E and F).

### 3.3 MUC5AC mRNA expression after intervention

**Figure 5:**
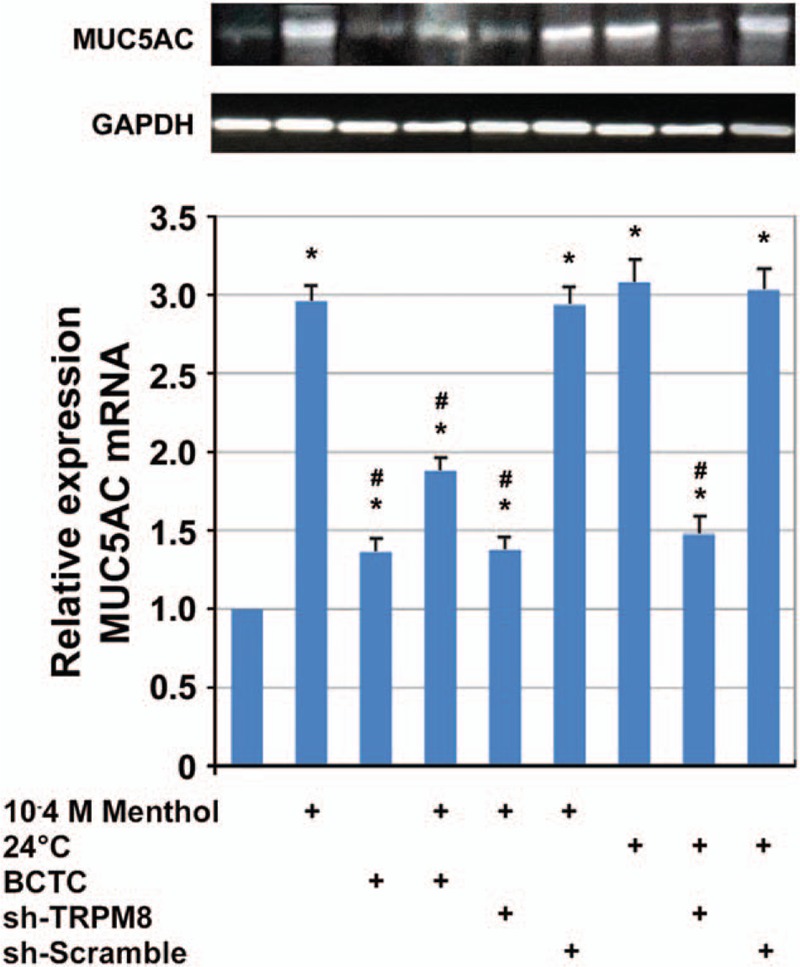
Relative quantitation of MUC5AC mRNA elicited by menthol/cold and effect of BCTC/TRPM8 shRNA. MUC5AC mRNA expression was evaluated using real-time PCR (N = 6). Data represent the fold increase in expression of MUC5AC relative to control. ^∗^*P* < .005 versus untreated control; #*P* < .005 versus cold/menthol-treated cells. PCR, polymerase chain reaction.

The elevation of MUC5AC mRNA expression is observed 4 hours after exposure to menthol and cold with a 2.96- and 3.08-fold (*P* < .001) increase, respectively. BCTC and TRPM8 shRNA reduced the menthol- and cold-induced MUC5AC mRNA expression (*P* < .001; Fig. 5).

### 3.4 Release of MUC5AC from HNE cells

**Figure 6:**
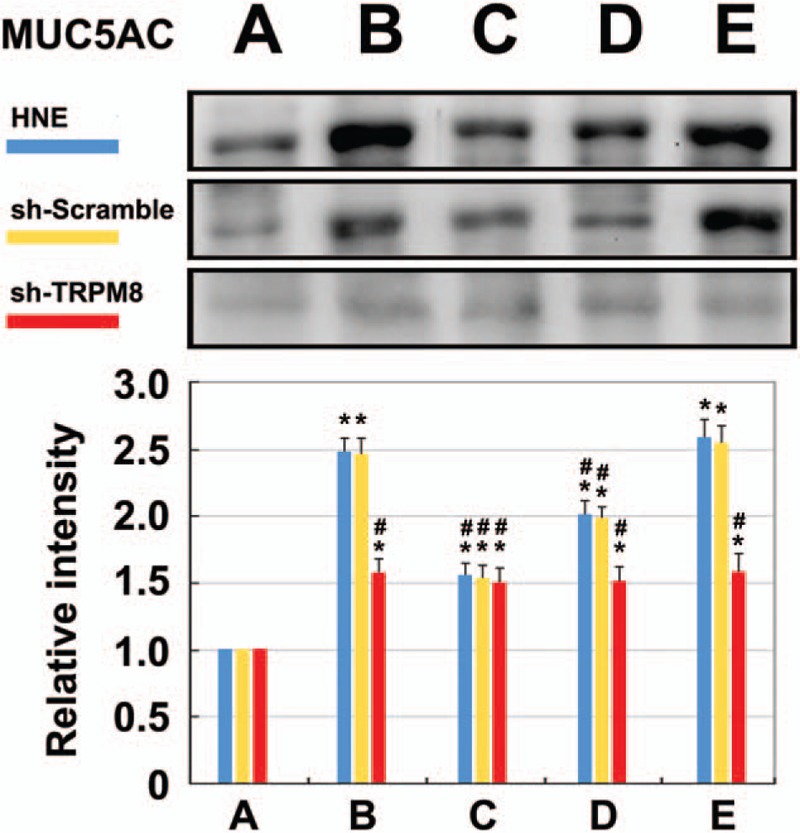
Levels of released mucin for HNE cells in each group, as measured by western blot analysis (N = 8): (A) control, (B) menthol (10^−4^ M), (C) BCTC (10^−4^ M), (D) menthol and BCTC, and (E) cold at 24°C. Both menthol (B) and cold at 24°C (E) induce a significant increase in the level of MUC5AC secretion, compared to the control group. The BCTC (D) has a statistically significant inhibitory effect on MUC5AC production that is induced by menthol (^∗^*P* < .005 versus untreated control; #*P* < .005 versus cold/menthol-treated cells).

Secreted MUC5AC in the cultured supernatant of the HNE cells is detected after 4 hours of exposure to test agents. Both menthol at 10^−4^ M and cold at 24°C induce a statistically significant increase in the level of MUC5AC secretion, with relative intensities that are respectively 2.48 ± 0.1 and 2.58 ± 0.14 times that for the control group. BCTC (10^−4^ M) and TRPM8 shRNA have statistically significantly attenuated the cold- and menthol-induced secretion of MUC5AC protein (Fig. 6).

## 4 Discussion

The idea of cold thermoreceptors in the nasal mucosa was proposed more than 20 years ago. Eccles and Jones found that a sensation of nasal patency was enhanced by breathing menthol vapor, even though there was no change in objective nasal resistance to airflow.^[13]^ Fontanati et al showed that nasal inhalation of cold dry air induces a nasopulmonary bronchoconstrictor reflex which is blocked by nasal local anesthesia in normal individuals.^[14]^ It is currently generally accepted that TRPM8 is predominantly expressed in a subpopulation of cold-responsive primary afferent sensory neurons within the trigeminal ganglia.^[15]^ TRPM8 is a thermosensor that is activated by cold temperatures of less than 28°C and cooling agents.^[16]^ Willatt and Jones showed that, for inspiratory temperatures ranging from 35°C to 25°C for the nasal septum, the subjective sensation of patency increases.^[17]^ The cooler the nasal lining, the clearer the nose feels. In the study by Keh et al, these cold receptors were thought likely to be TRPM8 that were observed in nerve fibers throughout the nasal subepithelium and which play an important role in the sensation of nasal airflow.^[12]^ The expression TRPM8 innervations in patients with allergic rhinitis show no significant difference from that for healthy subjects. This study demonstrates that TRPM8 is also highly expressed in nasal glandular tissue and epithelium. This study shows that TRPM8 is only partially expressed in blood vessels and that breathing menthol vapor does not affect the nasal mucosa blood flow, as measured by a laser Doppler method (data not shown). In conclusion, this study shows that the nasal secretory gland itself may act as both a cold-sensor and a secretory effector with the proposed physiological function that, in cold ambient temperatures, more nasal secretion is needed to humidify the inhaled cold dry air. It is also speculated that transient exposure to cold, such as the inhalation of cooling compound, may induce a brief neural response that evokes a feeling that the nose is clear. However, prolonged exposure to cold may induce mucus hypersecretion, which leads to rhinorrhea and decreased patency of the airway.

Dysfunctions of the upper and lower airways frequently coexist, since they are connected anatomically, physiologically, and immunologically.^[18]^ For example, coexistence of chronic allergic rhinitis with asthma is already widely recognized by clinicians as 1 airway, 1 disease. However, the study of bronchial epithelial cells is hampered by the difficulty of obtaining suitable samples, while the nasal cavity is a more accessible source of airway epithelial cells. Although Comer et al^[19]^ showed differences in inflammatory mediator release from paired nasal and bronchial epithelial cells from COPD patients, McDougall et al reported that despite discrepancies in soluble mediator levels, nasal cells can substitute in vitro bronchial epithelial cells in studies of lower airway inflammation because their responses to cytokine stimulation are similar.^[20]^ It has also been proposed that the serially passaged culture of primary cells might lose some characteristics of actual human nasal cells. In the study by Yoo et al, the first-passage cells were compared to their offspring, and it was found that passage-5 and passage-6 cells were swollen and lysed, which causes the cells to grow improperly.^[21]^ The authors suggested that cells up to passage 4 are suitable for following experiments. This study uses passage-3 and passage-4 cells and the results are quite consistent. It is also confirmed that the passaged culture system is applicable to drug screening and might also be a good platform for the etiology of airway disease.

In this study, the epithelial cells were obtained using a dispase-dissociation technique. Previous studies have demonstrated that this method maintains more *CK18* gene expression than a co-culture system.^[11]^ Briefly, the coculture technique cultures a mixture of epithelial cells and fibroblasts simultaneously. With the aid of a fibroblast, such as some growth factors, a faster cell growth rate and lower cost is achieved for epithelial cell culture. However, the authors experience is that the coculture technique produces a greater contamination rate since fibroblasts need medium with serum, but epithelial cells do not. Noruddin et al reported that a coculture system produces more basal and stem cell gene expression (CK14), but hinders the expression of *CK18* gene, which is the marker for ciliated and secretory epithelial cells.^[11]^ This situation jeopardizes this study since differentiated cell are tested in special conditions. Therefore, for the experiment that uses primary epithelial cells culture for pharmacotoxicological screening, a dispase-dissociation technique is used. Endres et al^[22]^ showed that the expression of CK18 genes continues to increase after 12 days, which demonstrates the progressive differentiation of the epithelial cells toward tissue formation.

Mucus hypersecretion, which is prominent in patients with allergic rhinitis and other chronic airway diseases, contributes to morbidity including airway plugging and recurrent infections. MUC5AC is usually the predominant mucin protein that is produced by airway goblet cells.^[23]^ This study shows that MUC5AC is released in the culture supernatant from viable HNE cells. Western blot analysis shows that cold (24°C) and menthol, which is a TRPM8 agonist, evoke prominent MUC5AC secretion in nasal epithelium cells. However, TRPM8 antagonist, BCTC, blocks the expression of MUC5AC. In terms of the immunohistochemical results, it is speculated that cold stimuli have direct effect on goblet cells, resulting in mucus production. The cold effects also lead to certain amines and peptides release from small granular cells and act in a positive feedback loop, promoting the secretion of mucus by goblet cells through paracrine mechanisms.^[24]^ In a study by Li et al, longer-term administration of cold or menthol is shown to induce mucus hypersecretion and to upregulate the expression of mucin genes, with a concomitant increase in the synthesis and secretion of mucins.^[8]^ The literature also shows evidence that epithelium and inflammatory cells in the airway, including eosinophils and T-lymphocytes, directly respond to temperature change.^[25]^ For a healthy subject, the production of mucus is important in airway antimicrobial defense and surface liquid homeostasis. However, activation of TRPM8 signal cascades in the airway epithelial cells in response to temporarily inhaled cold air could induce the excessive mucus production, which could exacerbate cold-induced allergic rhinitis or COPD. This study demonstrates the existence of these cold receptors in human nasal epithelium cells. The expression of TRPM8 in nasal epithelium cells might have some physiologic role for humans in cool environments, including secretion production and innate immune response.

## 5 Conclusion

This study demonstrates that TRPM8 (cold receptor) is present in nasal epithelium. When it is activated by moderate cooling at 24°C or menthol, TRPM8 induces the secretion of mucin. This study determines that TRPM8 channels are important regulators of mucin production. Therefore, TRPM8 antagonists could be used for the treatment of refractory rhinitis.
